# A comprehensive review of *Cornus officinalis*: health benefits, phytochemistry, and pharmacological effects for functional drug and food development

**DOI:** 10.3389/fnut.2023.1309963

**Published:** 2024-01-11

**Authors:** Wenhui Deng, Yuchen Liu, Yaodong Guo, Jie Chen, Hassan Idris Abdu, Muhmmad R. U. Khan, Chella Perumal Palanisamy, Jinjin Pei, A. M. Abd El-Aty

**Affiliations:** ^1^College of Physical Education, Shaanxi University of Technology, Hanzhong, China; ^2^Shaanxi Province Key Laboratory of Bioresources, QinLing-Bashan Mountains Bioresources Comprehensive Development C. I. C., Qinba State Key Laboratory of Biological Resources and Ecological Environment, College of Bioscience and Bioengineering, Shaanxi University of Technology, Hanzhong, Shaanxi, China; ^3^College of Health Management, Shangluo University, Shangluo, Shaanxi, China; ^4^ShaanxiUnion Research Center of University and Enterprise for Health Food Ingredient and Walnut Industry, Shangluo, Shaanxi, China; ^5^Pak-Austria Fachhochschule lnstitute of Applied Sciences and Technology, Haripur, Pakistan; ^6^Department of Chemical Technology,Faculty of Science, Chulalongkorn University, Bangkok, Thailand; ^7^Department of Pharmacology, Faculty of Veterinary Medicine, Cairo University, Giza, Egypt; ^8^Department of Medical Pharmacology, Faculty of Medicine, Atatürk University, Erzurum, Türkiye

**Keywords:** *Cornus officinalis*, health characteristics, biological activity, pharmacological effects, Cornaceae

## Abstract

**Introduction:**

*Cornus officinalis* sieb. et zucc, a deciduous tree or shrub, is renowned for its “Cornus flesh” fruit, which is widely acknowledged for its medicinal value when matured and dried. Leveraging *C. officinalis* as a foundational ingredient opens avenues for the development of environmentally friendly health foods, ranging from beverages and jams to preserves and canned products. Packed with diverse bioactive compounds, this species manifests a spectrum of pharmacological effects, including anti-inflammatory, antioxidant, antidiabetic, immunomodulatory, neuroprotective, and cardiovascular protective properties.

**Methods:**

This study employs CiteSpace visual analysis software and a bibliometric analysis platform, drawing upon the Web of Science (WOS) database for literature spanning the last decade. Through a comprehensive analysis of available literature from WOS and Google Scholar, we present a thorough summary of the health benefits, phytochemistry, active compounds, and pharmacological effects of *C. officinalis*. Particular emphasis is placed on its potential in developing functional drugs and foods.

**Results and Discussion:**

While this review enhances our understanding of *C. officinalis* as a prospective therapeutic agent, its clinical applicability underscores the need for further research and clinical studies to validate findings and establish safe and effective clinical applications.

## 1 Introduction

*Cornus officinalis*, also known as Fructus corni or dogwood, belongs to the Cornaceae family and can be found as a tree or shrub. It originated from the Caucasus region and subsequently spread across Turkey, Romania, Bulgaria, and other parts of continental Europe (1, 2). This species thrives in warm climates, with optimal growth occurring at temperatures between 20 and 30°C, while growth is hindered at temperatures exceeding 35°C. Dogwood flowers blossom during the spring season, and the fruits of the dogwood tree transform into a vibrant red color during autumn. The leaves of *C. officinalis* are arranged opposite to each other, resembling elm leaves, but they are more pronounced and lack serrations. The fruit of this plant is oval-shaped and possesses a sour taste.

**Figure 1:**
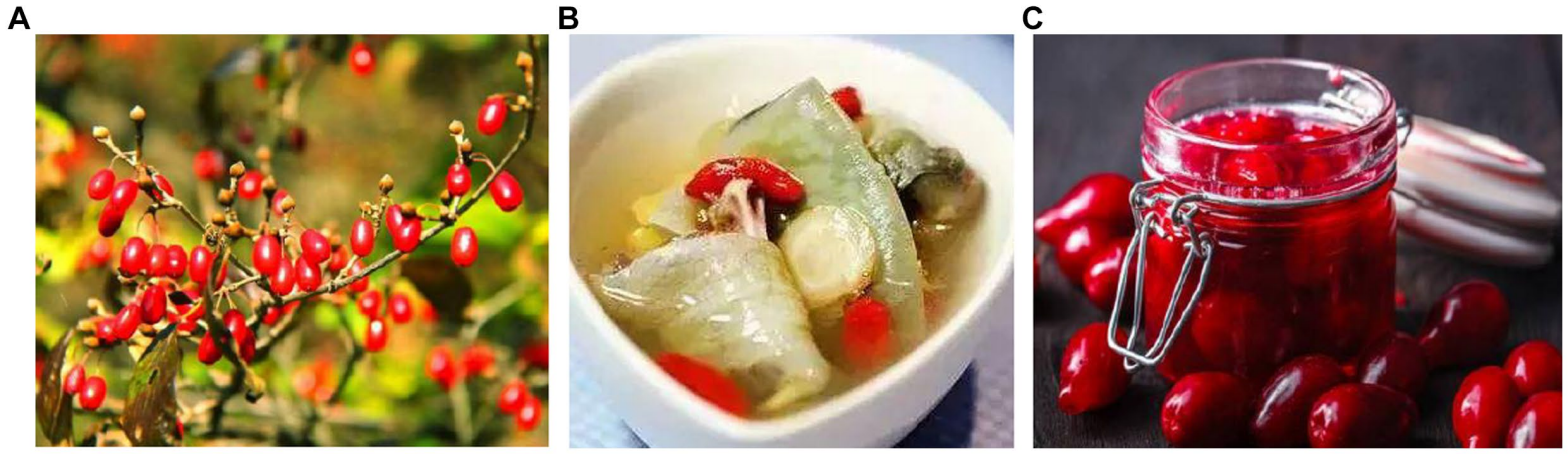
*Cornus officinalis* and its main products. **(A)** Fresh fruit; **(B)** dry fruit as functional seasoning; **(C)** Jam.

The growth of dogwoods can be categorized into four stages: the juvenile stage, early fruiting stage, fruiting stage, and senescence stage (3). *C. officinalis* holds a significant position in traditional Chinese medicine, boasting a rich history in China (4). It is one of the key components of “*Liu Wei Di Huang Wan*” and contains various beneficial substances, such as ursolic acid, gallic acid, malic acid, saponins, phenols, resins, vitamin A, vitamin C, and others (5, 6). These components contribute to its biofunctionality, which includes safeguarding the cardiovascular system, boosting the immune system, and exhibiting anti-inflammatory, antibacterial, and antioxidant properties, as well as aiding in lowering blood lipids and enhancing human memory. Despite the abundance of active substances in *C. officinalis* and its positive effects on the body, it possesses a sour taste. The primary forms in which *Cornus officinalis* is consumed include dried fruit, used as a traditional functional seasoning, and jams (Figure 1). While *C. officinalis* holds considerable nutritional value, further research and development in the food industry are still needed. This review aims to elucidate the biological activity, health benefits, and potential applications of *C. officinalis* in food and medicine while also providing an overview of the current research status and future prospects for *C. officinalis*.

## 2 Literature analysis of *Cornus officinalis* research

CiteSpace software, developed by Dr. Chenchaomei at Redsell University in the United States, is a powerful visual analysis tool designed to explore the multidimensional, temporal, and dynamic aspects of scientific literature (7–10). In this study, the Web of Science (WOS) database serves as the primary source of information. The CiteSpace analysis tool (11, 12) was employed to investigate the current developmental status and identify the research hotspots related to *C. officinalis*. Herein, we utilized the Web of Science (WOS) database as the retrieval platform and conducted a literature search using “*Cornus officinalis*,” “ingredients,” or “activities” or “pharmacology” as the keyword. The search encompassed papers published between 2013 and 2023. Following the screening process, a total of 233 papers focusing on the bioactivity of *C. officinalis* were obtained. The selected literature was exported and saved for further analysis. CiteSpace software was employed to study and cluster the authors and keywords. To gain deeper insights into the research field of *C. officinalis*, we utilized both the WOS platform^1^ and the bibliometric analysis platform^2^ (13–15). By leveraging these platforms, we examined and analyzed the collaborative relationships and the number of published documents within the field of *C. officinalis* research (14, 16, 17).

### 2.1 Analysis of annual document issuance

**Figure 2:**
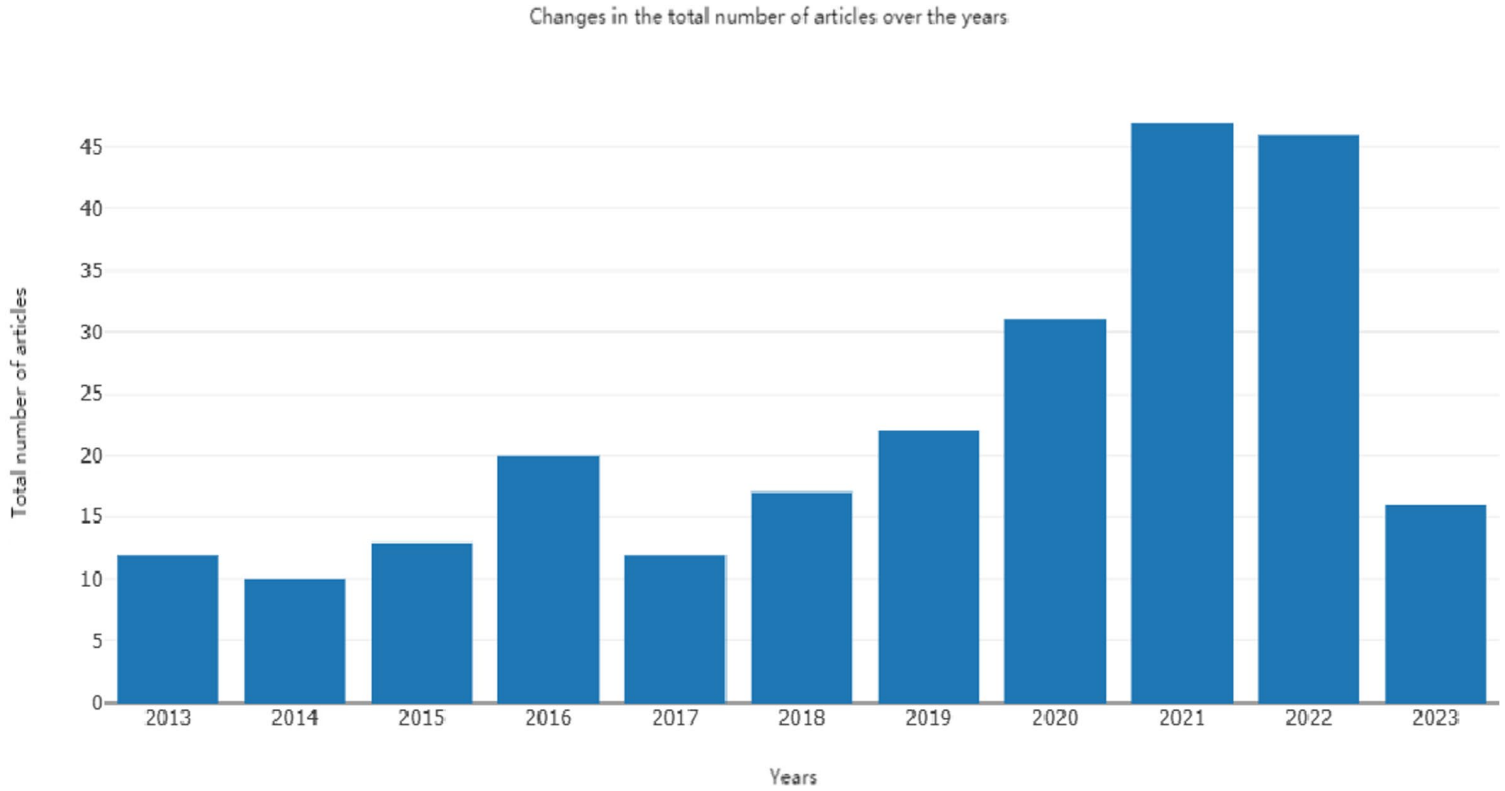
Regression analysis of the annual document volume in the WOS database.

The annual publication count serves as an indicator of the developmental trajectory and dynamics within a specific research field, providing insights into its level of popularity and interest (18). In Figure 2, we present the fitted and analyzed data showing the number of publications focused on the bioactivity of *C. officinalis* research from 2013 to 2023. Since 2017, there has been a notable increase in the core literature on *C. officinalis*, driven by a growing demand for health products and the expanding herbal medicine sector. However, in 2023, there appears to be a relative decline in the number of publications on dogwood. This decline could be attributed to the extensive research conducted in recent years on the chemical constituents and pharmacological effects of *C. officinalis*, leading to a comprehensive understanding of its biological properties both domestically and internationally. Consequently, there has been a relative deceleration in *C. officinalis* research post-2023. Additionally, in 2023, the primary production region of dogwood experienced extreme weather conditions, with temperatures dropping from 25 to 28°C to 1–3°C below zero. This climatic shift resulted in a reduction in dogwood production, significantly impacting the overall national output. The study’s findings shed light on the current status of *C. officinalis* research and its future outlook.

**Figure 3:**
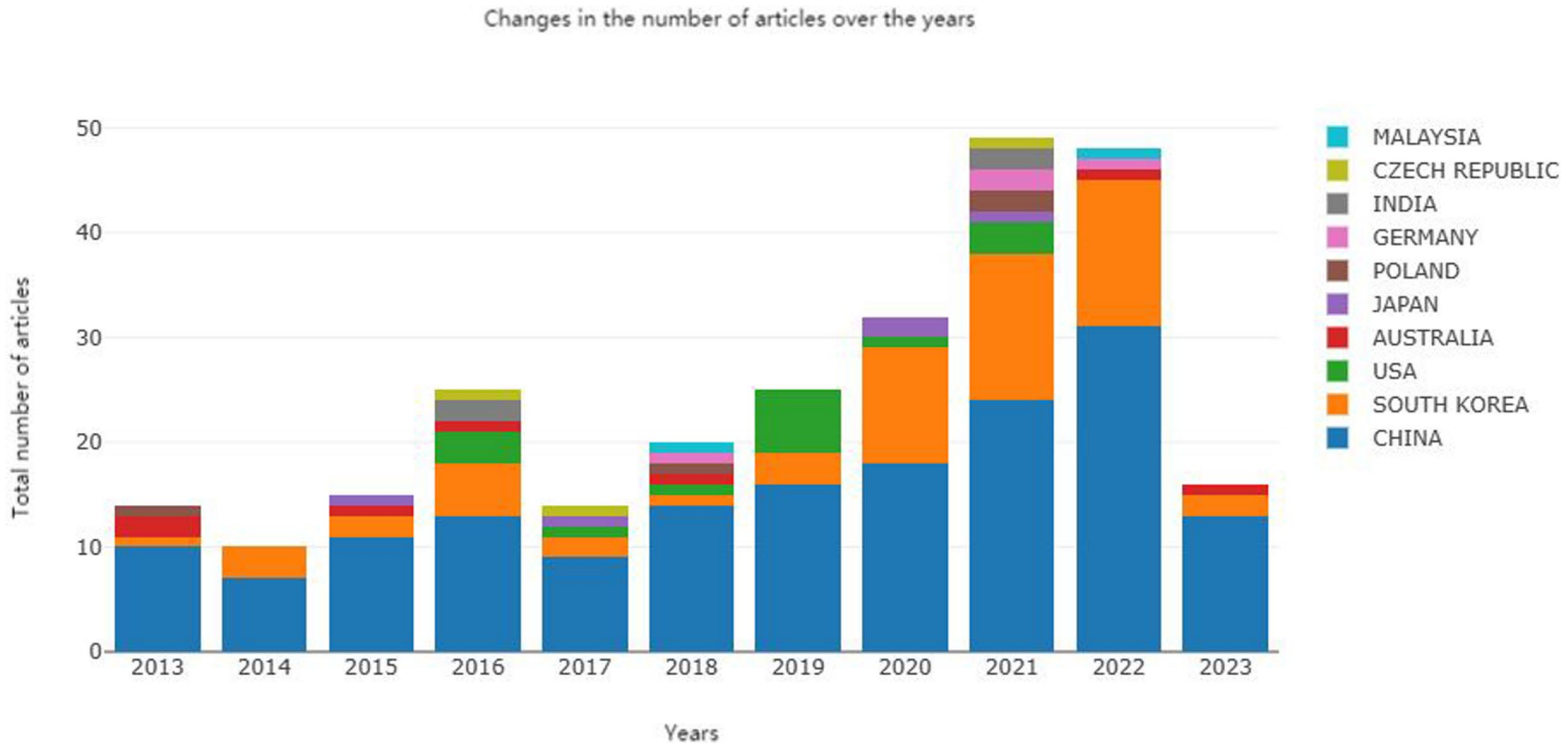
Analysis of worldwide document publication trends from 2013 to 2023.

Through statistical analysis of the global publication count on the bioactivity of *C. officinalis*, as well as the publication counts from different countries, notable findings emerged. As illustrated on the right side of Figure 3, the top 10 countries in terms of publication volume during this decade were China, South Korea, the USA, Japan, Poland, Germany, Australia, India, the Czech Republic, and Malaysia. Notably, China had a significantly higher number of publications than the second-ranked countries, South Korea and the United States. From 2013 to 2020, the number of publications on the bioactivity of *C. officinalis* remained relatively stable. However, a substantial increase was observed from 2021 to 2023, primarily driven by China and South Korea. Particularly in 2021, there was a sudden surge in the number of publications related to *C. officinalis* bioactivity, with China being the primary contributor. Over the past 10 years, China has demonstrated rapid and prominent development in *C. officinalis* bioactivity research, consistently leading the field and making significant contributions.

### 2.2 Analysis of published periodicals

**Figure 4:**
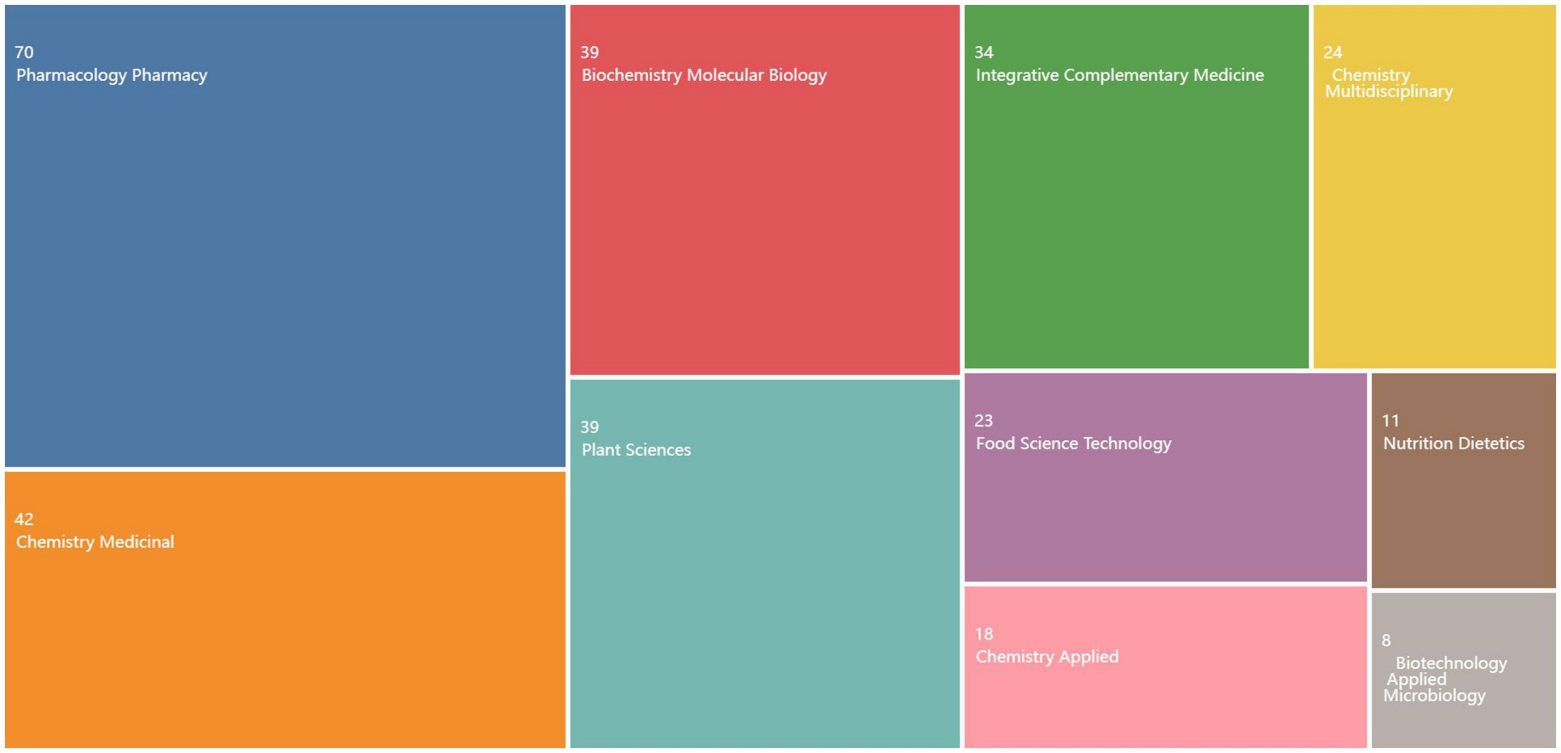
Analysis of global journal publications on the bioactivity of *C. officinalis* from 2013 to 2023.

The research focus in this field can be inferred from the volume of literature published in relevant journals (19). Analysis of the industry fields of the selected documents in the WOS database (Figure 4) reveals that the research direction primarily lies within the domains of pharmacology, pharmacy, medicinal chemistry, and plant sciences. Table 1 presents the analysis of selected journals obtained through the bibliometric online analysis platform. The top three journals in terms of publication volume are JOURNAL OF ETHNOPHARMACOLOGY, FRONTIERS IN PHARMACOLOGY, and MOLECULES. The dominance of these journals, along with other top-ranking journals in terms of publication volume, reflects the significant academic influence of *C. officinalis* bioactivity research within leading national journals. Notably, the most published and cited paper is from the JOURNAL OF ETHNOPHARMACOLOGY. Although the study of *C. officinalis* bioactivity has gained international recognition, there is still ample room for further research in this field. This holds great significance for the development of the *C. officinalis* industry.

**Table 1:** Analysis of journal publications from 2013 to 2023.

Journal name	Total number of articles	Total cited times	Average cited times
Journal of Ethnopharmacology	16	152	9.50
Frontiers in Phapmacology	14	48	3.43
Molecules	13	31	2.38
Evidence-based Complementary and Alternative Medicine	7	7	1.00
International Journal of Molecular Sciences	7	2	0.29
Phytotherapy Research	4	41	10.25
Nutrients	4	22	5.50
Natural Product Research	4	17	4.25
International Journal of Biological Macromolecules	4	9	2.25
Industrial Crops and Products	4	4	1.00

### 2.3 Analysis of hotspot research

**Figure 5:**
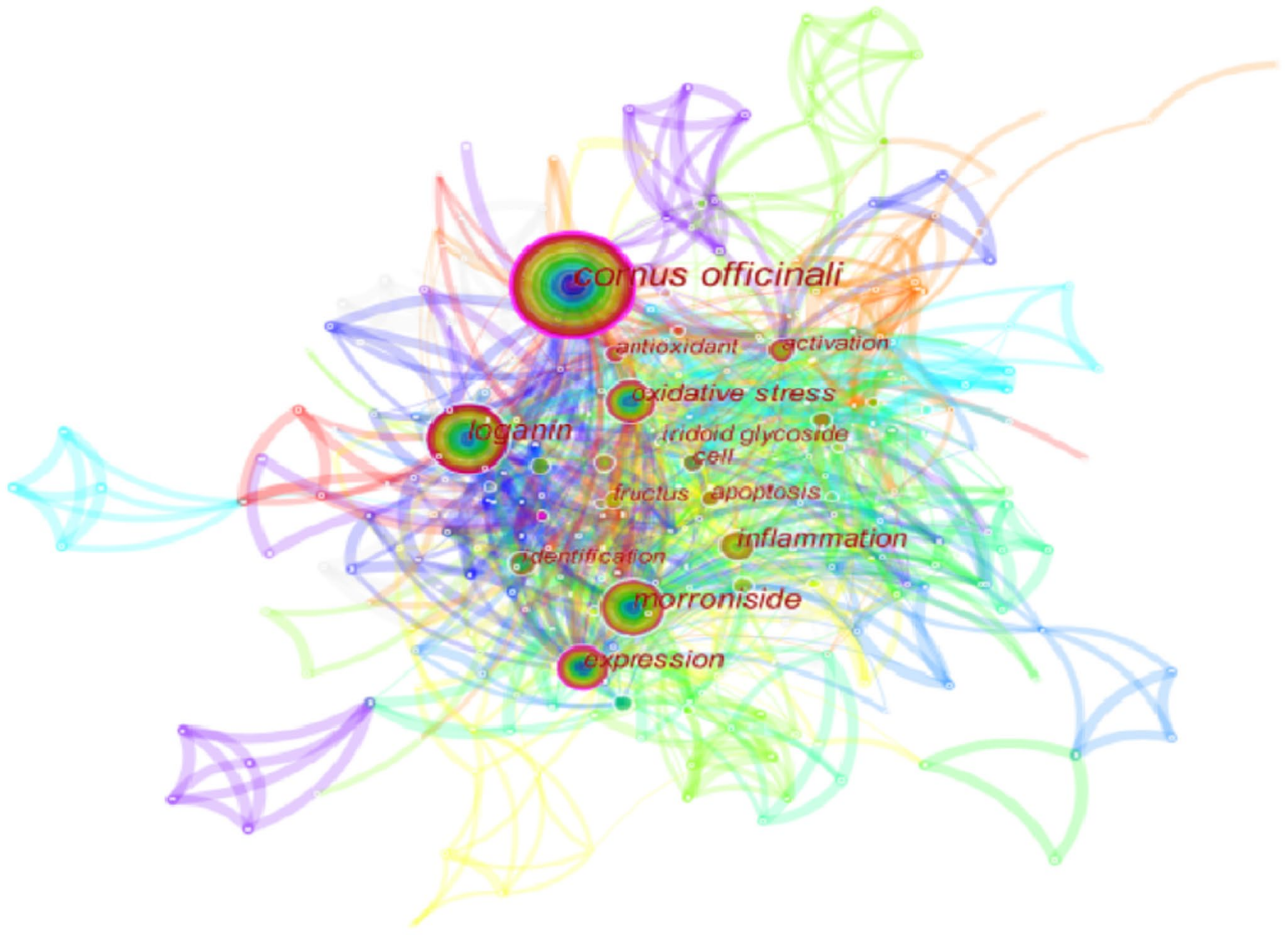
Node map of keywords in the bioactivity of *C. officinalis*.

This section examines the research hotspots in the field of biological activity of *C. officinalis* by analyzing the keywords used in scholarly studies. The visualization of these keywords is presented in Figure 5. The identified keywords related to the biological activities of *Cornus officinalis* included “loganin,” “morroniside,” “expression,” “oxidative stress,” “inflammation,” “iridoid glycoside,” “fructus,” “identification,” “activation,” and “cell,” in addition to the keyword “*Cornus officinalis*” itself. This progressive exploration of the biological activity of *C. officinalis* holds significant importance for the development of the *C. officinalis* industry.

**Figure 6:**
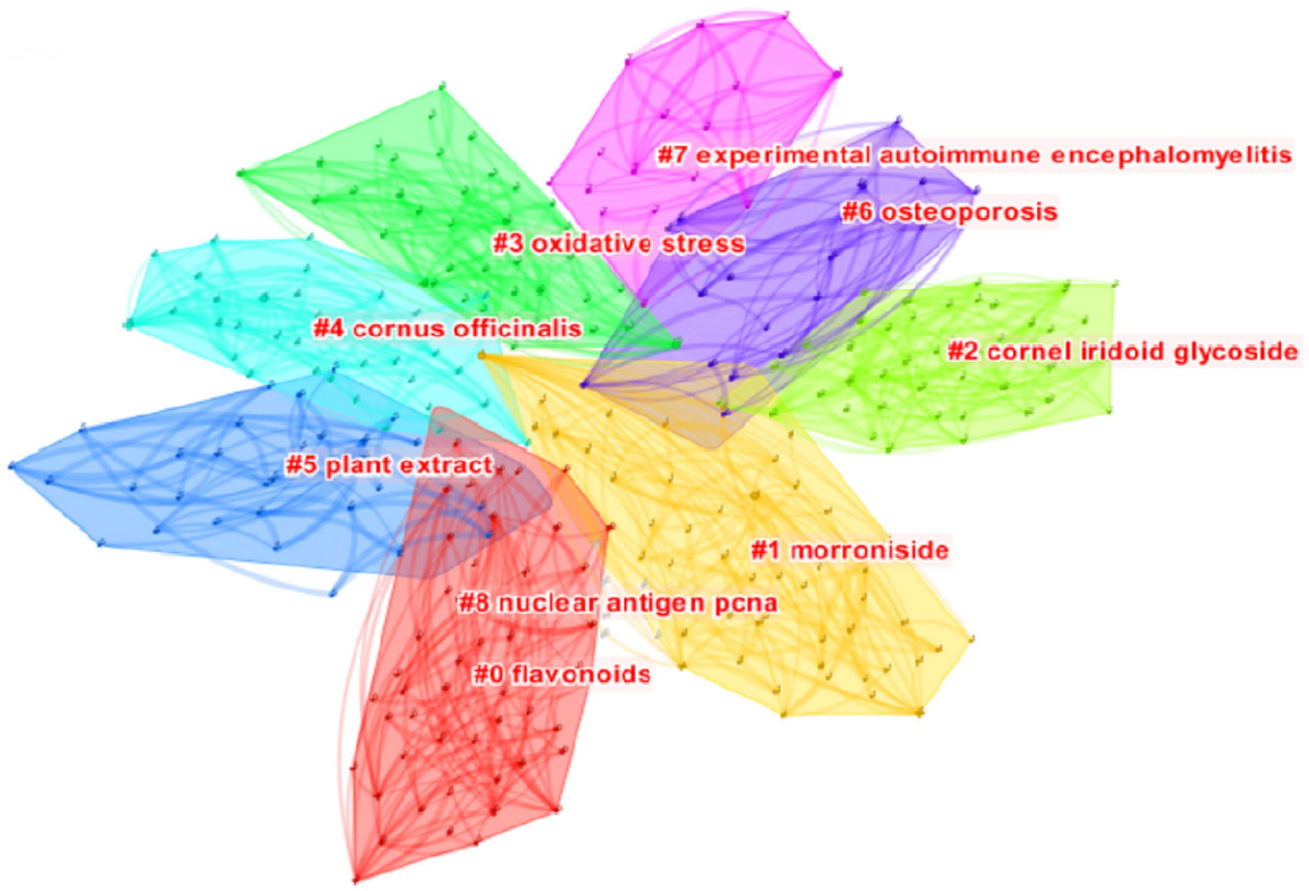
Clustering map of bioactivity for *Cornus officinalis*.

Figure 6 presents the cluster analysis of bioactive keywords related to *C. officinalis* in the WOS database. This analysis helps to elucidate the interrelationships among keywords. The clustering modules, as depicted in the figure, reveal that research on the biological activity of *C. officinalis* has evolved from plant extracts to encompass oxidative stress, morroniside, cornel iridoid glycoside, flavonoids, and other activities and mechanisms associated with *C. officinalis* products. This signifies a growing depth of research in this field.

**Figure 7:**
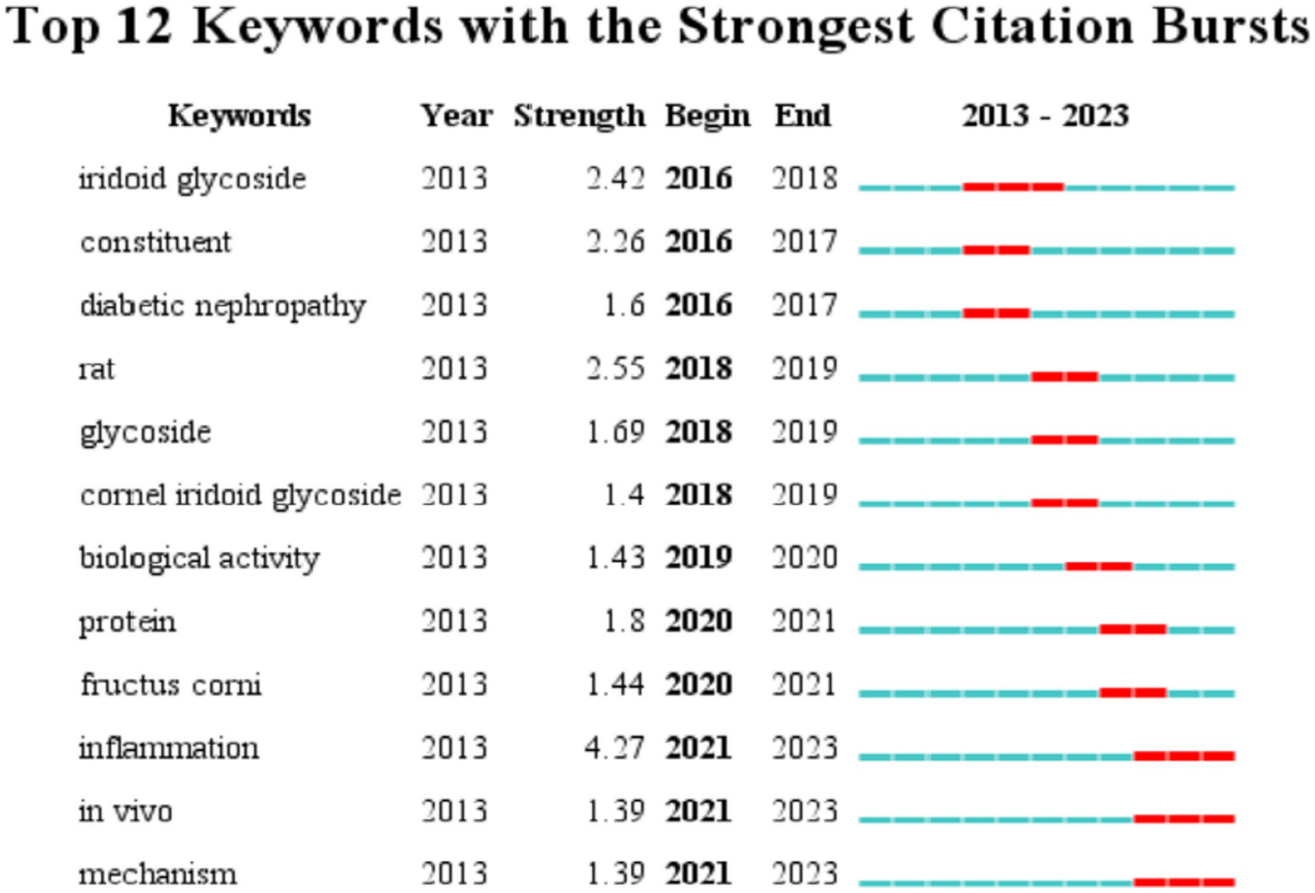
Keyword emergence map.

Figure 7 presents an analysis of keyword occurrences in the WOS database pertaining to the bioactivity study of *C. officinalis*. The frequency of keywords provides insights into the evolving research focus over time. In earlier periods, there was a greater emphasis on investigating the composition of *C. officinalis*. However, starting in 2016, keywords such as iridoid glycoside and constituent gradually gained prominence. This suggests that research on *C. officinalis* extends beyond its basic components and encompasses the study of specific active substances within the plant. From 2019 onward, there was a notable increase in keywords such as protein, inflammation, and biological activity, indicating a deeper exploration of the diverse bioactivity of *C. officinalis*. This shift signifies a more comprehensive investigation into the potential medicinal properties of the plant.

## 3 Classification and distribution

*Cornus officinalis*, a species native to the northern United States, encompasses various subspecies, including European Cornus (*C. mas*), Japanese Cornus, Chinese Cornus (*C. officinalis*), and Pacific Cornus (*C. nuttallii*). In China, *C. officinalis* has a wide distribution, with areas such as Lin’an County in the Tianmu Mountains region of northwestern Zhejiang Province (30 N, 119E), Nanyang County in the Fuyu Mountains region of southwestern Henan Province (33 N, 112E), and Hanzhong County in the Qinlingba Mountains region of southwestern Shaanxi Province (32 N, 107E) (20–23). It is also found in certain regions of Japan and South Korea (24, 25). The variation in environmental factors such as climate, soil composition, temperature, and humidity contributes to differences in the content of active substances, pulp quality, and even taste of *C. officinalis* across different regions. These active substances play a crucial role in the pharmacological effects of *C. officinalis*, leading to varying outcomes in different geographical areas (22). Recognizing its unique biological activity and pharmacological effects, China has designated *Cornus officinalis* as a medicinal plant under national protection (23, 26).

Kingdom: Plants.

Phylum: Angiospermae.

Class: Monocotyledons.

Tribe: Cornus.

Suborder: Cornus.

Order: Cornus.

Family: Cornus.

Genus: Cornus.

Species: *Cornus officinalis.*

Distribution: China, Korea, USA, Japan.

## 4 Phytochemistry of *Cornus officinalis*

Ongoing investigation into *C. officinalis* has unveiled a multitude of bioactive substances with promising health advantages. Comprehensive research has identified hundreds of active compounds derived from different plant components (Table 2 provides details on some of these compounds).

**Table 2:** Phytochemical constituents isolated from *C. officinalis.*

Compound	Content	References
Loganin	C_17_H_26_O_10_	(27)
Morroniside	C_17_H_26_O_11_	
7-dehydrologanin	C_17_H_24_O_10_	(28–30)
7-*O*-butylmorroniside	C_21_H_34_O_11_	
10-hydroxyhastatoside	C_18_H_28_O_11_	
β-dihydrocornin	C_17_H_26_O_10_	
Cornuside	C_24_H_30_O_14_	
Mevaloside	C_12_H_20_O_8_	
Soyasaponin VI	C_54_H_84_O_21_-	
2,2-Dimethylcyclohexane-1,3-dione	C_8_H_12_O_2_-	
Gallic acid	C_7_H_6_O_5_	
Alphitolic acid	C_30_H_48_O_4_	(31)
Urlosic acid	C_30_H_48_O_3_	
Ellagic acid	C_14_H_6_O_8_	
5-hydroxymethyl-2-furfural	C_6_H_6_O_3_	(32)
1,2,3,6-tetragalloyl-β-D-glucose	C_34_H_28_O_22_	
Quercetin3-*O*-β-D-Glucuronide	C_21_H_18_O_13_	
Tellimagrandin I	C_34_H_26_O_22_	(33)
Tellimagrandin II	C_41_H_30_O_26_	
1,2,3,4,6-pentagalloyl-β-Dglucopyranose	C_41_H_32_O_26_	
8-hydroxy-10-hydrosweroside	C_16_H_24_O_10_	(34)
3-*O*-*β*-galactopyranoside	C_21_H_21_O_11_	(35)
Cyanidin 3-*O-β*-galactopyranoside	C_21_H_21_ClO_11_	
Pelargonidin 3-*O-β*-galactopyranoside	C_22_H_23_ClO_11_	
7-*β-O*-ethylmorroniside	C_19_H_30_O_11_	(36)
7-*α-O*-ethylmorroniside	C_19_H_30_O_11_	
3-*O*-caffeoylquinic acid *n*-butyl ester	C_16_H_18_O_9_	
vomifoliol	C_13_H_20_O_3_	
2,3-Di-*O*-galloyl-D-glucose	C_20_H_20_O_10_	(37)
1,2,3-tri-*O*-galloyl-β-D-glucose	C_27_H_24_O_18_	
1,2,6-tri-*O*-galloyl-β-D-glucose	C_27_H_24_O_18_	
1,2,3,6-tetra-*O*-galloyl-β-D-glucose	C_34_H_28_O_22_	
Quercetin-3-*O*-glucoside	C_21_H_20_O_12_	(38)
4-HydroxyBenzeneethanol	C_8_H_10_O_2_	(39)
Glutaconic-anhydride	C_5_H_4_O_3_	
Methyl 2-hydroxybenzonate	C_8_H_8_O_3_	

Initially, the edible part of *Cornus officinalis* primarily consisted of fruit pulp (40). However, ongoing research has revealed the presence of varying levels of active substances in different parts of the plant. For instance, a study identified three active compounds, namely, 3,3′-di-*O*-methylellagic acid 4-(5″-acetyl)-α-l-arabinofuranoside, 6α-dihydrocornic acid, and 6β-dihydrocornic acid, extracted from the roots of *C. officinalis* (41, 42). Flavonoid substances such as β-sitosterol, glucose, and sucrose were extracted from the leaves (43, 44), and the ethanolic extract of the leaves yielded approximately 30–40 active substances, including phenols, esters, ketones, tannins, and organic acids (as listed in Table 3) (38, 51). Other studies have focused on the leaves, resulting in the extraction of three new iridoids, one of which exhibited inhibitory effects on the lung cancer cell line A-549 (52). Moreover, five substances with α-glucosidase inhibitory activity were isolated from the aqueous extract of *C. officinalis* fruits, named cornucadinoside A-E (1–5) (53). Additionally, research on *C. officinalis* seeds led to the isolation of eight substances, including various galloyl-glucose compounds and phenolic acids (54–59). Ursolic acid, obtained from the seeds or peels of *C. officinalis*, showed nontoxic properties in LD50 tests and single-cell gel electrophoresis (60, 61). While the majority of active substances are found in the fruit, studies have also identified new compounds, such as cornuside, cornusiin G, methyl malate, and sedoheptulose gallate, highlighting the presence of dimeric hydrolyzable tannins and other molecular components (62, 63). Furthermore, the fruits of *C. officinalis* yielded a new bisiridoid glucoside named cornutide, as well as 7β-O-dimethyl butanedioate morroniside and caffeoyltartaric acid dimethyl ester, which were structurally characterized using various spectroscopic analyses (64, 65).

**Table 3:** Nutrient composition and content in *C. officinalis.*

Sample	Element	Units	Content	References
Minerals	Fe	ug/g	43	(45, 46)
	Ca	ug/g	1750	
	Mg	ug/g	3,390	
	Pb	ug/g	0.08	
	As	ug/g	0.003	
	Mn	ug/g	1.6	
	Al	ug/g	3.5	
	Cu	ug/g	8.7	
	Zn	ug/g	6.6	
	Se	ug/g	0.007	
	Co	ug/g	0.0009	
	Sr	ug/g	0.004	
	Na	ug/g	0.03	
	K	ug/g	0.10	
Amino acids	Aspartic acid	mg/kg	2625.41	(47–49)
	Serine	mg/kg	1138.34	
	Glutamic acid	mg/kg	1341.20	
	Threonine	mg/kg	697.35	
	Glycine	mg/kg	442.63	
	Alanine	mg/kg	611.25	
	Proline	mg/kg	98.25	
	Valine	mg/kg	328.30	
	Isoleucine	mg/kg	226.40	
	Leucine	mg/kg	685.41	
	Tyrosine	mg/kg	142.01	
	Phenylalanine	mg/kg	147.02	
	Lysine	mg/kg	216.43	
	Histidine	mg/kg	551.60	
	Arginine	mg/kg	106.76	
Vitamins	Vitamin C	mg/kg	1840.5	(45, 50)
	Vitamin E	mg/kg	100.2	
	Vitamin B2	mg/kg	785.4	
	Vitamin B12	mg/kg	237.4	

## 5 Bioactive compounds in *Cornus officinalis*

### 5.1 Cyclic enol ether terpene glycosides in *Cornus officinalis*

Cyclic enol ether terpene glycosides are widely distributed in nature, primarily in dicotyledonous plants. Various herbal medicines, including Dihuang, Xuan Shen, Strychnos, Lonicera, and Gentian, contain cyclic enol ether glycosides (66, 67). Cyclic enol ether terpenes encompass different structural types, such as cyclic enol ether glycosides, cleaved cyclic enol ether terpene glycosides, and cyclic enol ether terpene esters. Cyclic enol ether terpene glycosides in *C. officinalis* are shown in Table 4 (75).

**Table 4:** Classification of cyclic enol ether terpene glycosides in *C. officinalis.*

**Type**	**Compound**		**References**
Cyclopentane-type cyclic enol ether Terpene	Verbascoside	Determination of content by HPLC-DAD; Determination of content by HPLC-DAD-TOF-MS (65, 68)	(69, 70)
	Strychnoside		
	7-Dehydrostrychnoside		
Cleaved ring cyclic enol ether terpenes	7-Monosidine		(28, 57, 71–73)
	7-*O*-Methylmonosidine		
	7-O-Ethylmonosidine		
	7-O-Butylmonosidine		
	Dehydromonoside		
	Cornuside		
	Cornuside III		
	Cornuside IV		
	Strychnine		
	Strychnoside		
	Sweretin		
Bicyclic enol ether terpenes	Cornuside II		(74)

**Figure 8:**
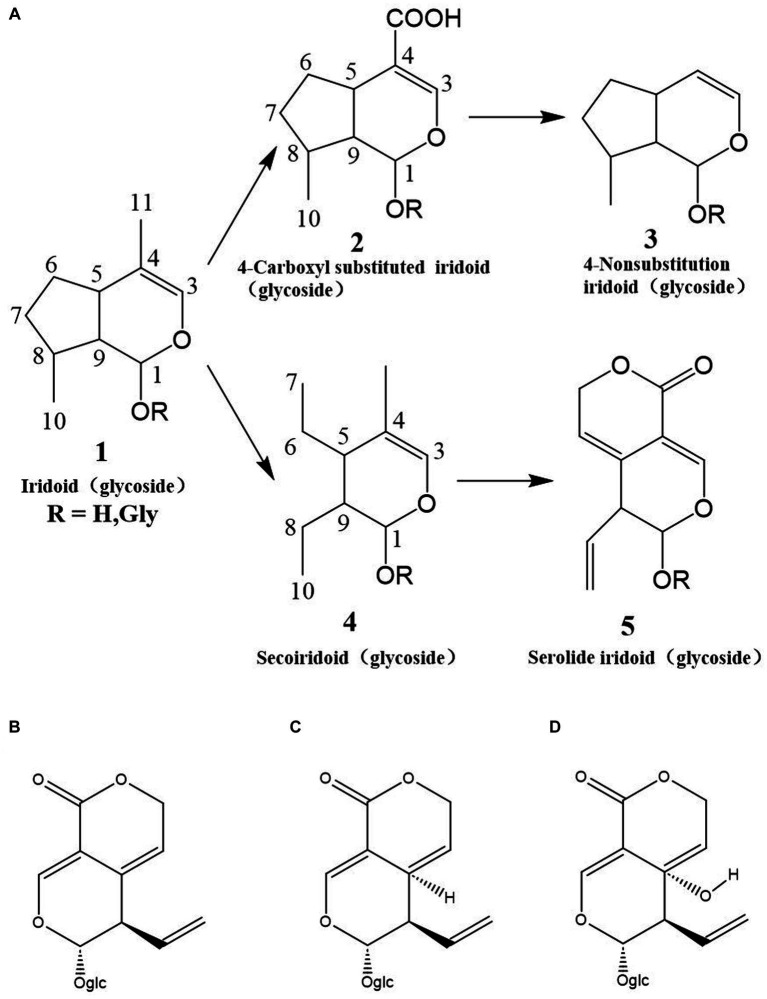
Classes of cyclic enolether glycosides and their interaction relationships. **(A)** Classification workframe; **(B)** Gentiopicri; **(C)** Sweroside; **(D)** Swertiamarin.

Cyclic enol ether glycosides are highly abundant bioactive compounds found in various parts of *C. officinalis*, including the fruit, peel, shell, and branches. Researchers have employed different extraction methods to isolate these compounds from *C. officinalis* (76). In one study, dried fruits were crushed and subjected to methanol extraction using an ultrasonic extraction method. The crude extract was further purified by passing it through macroporous resin to eliminate impurities such as pigments, resulting in a substantial yield of cyclic enol ether terpene glycosides. Another approach involved microwave irradiation for the extraction of these glycosides from *C. officinalis*. The optimized conditions included using 72% ethanol as the solvent, a liquid-to-material ratio of 15 mL/g, 10 min of microwave power at 400 W, and two consecutive extraction cycles. These extraction methods have proven effective in obtaining high concentrations of cyclic enol ether glycosides from *C. officinalis*. As research has progressed, a diverse array of cyclic enol ether terpene glycosides have been identified and extracted from *C. officinalis*. Among them are loganin, sweroside, morroniside, kingiside, loganic acid, 10-hydroxycornin, 7-ketologanin, 7-*O*-methylmorroniside, cornuside I, 8-epikingiside, secologanoside, 10-hydroxyhastatoside, hastatoside, dihydrocornin, swertimarin, secologanin, cornin, 7-*O*-methylmorroniside, cornuside II, and dehydromorroniside aglycone. These compounds represent a diverse range of cyclic enol ether terpene glycosides derived from *C. officinalis* (77–82) (Figure 8).

Morroniside, derived from the medicinal plant *C. officinalis*, is an atypical secoiridoid that possesses a unique six-membered cyclic endoether fragment. This compound belongs to the class of iridoid glycosides and has been found to exhibit potent antioxidant properties (83). In studies investigating the effects of morroniside on cytotoxicity induced by hydrogen peroxide in human neuroblastoma tumor SH-SY5Y cells, it was observed that morroniside effectively reduced intracellular calcium accumulation, mitigated hydrogen peroxide-induced mitochondrial membrane potential (MMP) disruption, and decreased the percentage of apoptosis triggered by hydrogen peroxide (84). Loganin, extracted from the fruit of *C. officinalis*, was evaluated in male mice subjected to destabilization of the medial meniscus of the right knee (DMM). Mice were treated with loganin at concentrations of 30 or 100 μg/mL injected into the osteoarthritic space for a period of 2–3 months. The results demonstrated that loganin exhibited a protective effect by slowing the progression of osteoarthritis, highlighting its potential for the treatment of this condition (83). The impact of cornelian cyclic enol ether terpene glycosides on platelet aggregation and bleeding time has been investigated in animal studies involving mice and rabbits. The findings revealed that these glycosides effectively inhibited platelet aggregation induced by adenosine diphosphate (ADP) both *in vitro* and *in vivo*. Moreover, the effects on bleeding time were examined using a tail break assay in mice, which demonstrated that high doses of cornelian cyclic enol ether terpene glycosides had the ability to prolong bleeding time in rats (85).

### 5.2 Flavonoids

Flavonoids are a class of phenolic compounds that are widely distributed in various plants, including flowers and vegetables. They are considered plant secondary metabolites and are characterized by their C6-C3-C6 structure (86, 87). This structure consists of two benzene rings (A and B rings) interconnected by a central three-carbon chain, and the C3 part can either form a six-membered ring with the C6 part or be a lipid chain. Flavonoids encompass a diverse group of compounds, and they are generally categorized into flavones and flavonols based on the basic structure of their parent nucleus. Additionally, there are other variations and derivatives of flavonoids, such as isoflavones, which have their B ring attached to the C3 position, and they can be further divided into isoflavones and dihydroisoflavones.

Flavonoids encompass a variety of compounds, including well-known subclasses such as anthocyanins, proanthocyanidins, isoflavones, and catechols. These compounds are commonly found in nature as glycosides or with carbon glycosyl groups attached to sugars. Flavonoids possess significant medicinal value and have been associated with benefits such as improving cardiovascular health, preventing arterial apoptosis, reducing the risk of cancer, and promoting overall well-being. Furthermore, they exhibit diverse bacteriostatic effects against various microorganisms, including gram-positive bacteria, gram-negative bacteria, and fungi (88, 89). Among the subclasses of flavonoids, flavones are particularly noteworthy. They are characterized by two phenolic hydroxyl groups on the benzene rings (A and B) connected by a three-carbon atom, forming a fundamental parent nucleus known as 2-phenylchromone. Flavonols, on the other hand, contain ketone groups. Some examples of flavonols include kaempferol, quercetin, prunetin, and fisetin. Foods such as apples, tomatoes, and kale are abundant sources of flavonols (90).

*Cornus officinalis*, including its fruits and leaves, is a rich source of flavonoids, which exhibit notable properties in terms of tumor inhibition and antioxidant activity. This characteristic makes *C. officinalis* highly promising for pharmacological development (91, 92). In one study, flavonoids were extracted from dried *C. officinalis* using a direct warm immersion method, where 70% ethanol was added to the plant material. The extraction process involved a constant water bath at 80°C for 2 h, followed by cooling and filtration. Another investigation explored the use of ultrasonic treatment with 70% ethanol at 80% power and 60°C for 20 min to extract flavonoids. The total flavonoid content in *C. officinalis* was determined by adding 1 mL of the test sample to a test tube, followed by the addition of 10 mL of diethylene glycol reagent and 1 mL of NaOH. The mixture was vigorously shaken and allowed to react in hot water at 37°C for 60 min. Finally, the absorbance at 420 nm was measured, and a standard curve was prepared (93).

An investigation focused on the activity-guided isolation of an 80% acetone extract from *C. officinalis,* which resulted in the identification of various flavonoids, including catechin, quercetin-3-*O*-β-D-glucuronide, quercetin-3-*O*-β-D-glucopyranoside, and kaempferol-3-*O*-β-D-glucopyranoside (94). Furthermore, several other flavonoids have been isolated from *C. officinalis*, such as kaempferol-3-*O*-β-D-galactoside, quercetin, rutin, quercetin-3-*O*-a-L-rhamnosyl-(1–6)-β-D-galactoside, and kaempferol-3-*O*-a-L-rhamnosyl-(1→6)-β-D-glucoside. These bioactive compounds are extracted from *C. officinalis* and exhibit promising potential for human health development.

A study was conducted to evaluate the antibacterial and antioxidant properties of flavonoids extracted from *C. officinalis*. The results demonstrated a significant impact of these flavonoids on inhibiting the activity of Salmonella (95). Moreover, the DPPH radical scavenging rate and hydroxyl radical scavenging rate of *C. officinalis* leaf flavonoids at a concentration of 1.2 mg/mL exceeded 75%, indicating strong antioxidant activity. The study also identified three different flavonoids, namely, 5-hydroxy-6,7,8,3′,4′,5′-hexamethoxyflavon-3-ol, demethyldigicitrin, and quercetin, extracted from the ethanolic extract of *C. officinalis*. Their EC_50_ values were measured as 2.74 ± 0.10, 3.41 ± 0.09, and 1.27 ± 0.25 μg/mL, while their IC_50_ values were 27.91 ± 0.18, 28.92 ± 0.12, and 81.38 ± 0.33 μg/mL, respectively. These findings provide empirical evidence supporting the medicinal potential of *C. officinalis* (96).

### 5.3 Polysaccharide

One of the primary active constituents of *C. officinalis* is polysaccharides (97). The dehydration and condensation of several monosaccharides lead to the formation of macromolecular polysaccharides, which are prevalent and significant in nature. In specific studies, *C. officinalis* polysaccharides have been modeled using artificial neural networks (ANN) and optimized through the application of genetic algorithms coupled with ANNs (GA-ANN). The optimal extraction parameters for *C. officinalis* polysaccharides were determined, and statistical techniques demonstrated that ANN could accurately estimate the yield of *C. officinalis* polysaccharides. The settings that yielded the best results (7.85 ± 0.09%) were as follows: 350 W of ultrasonic power, an extraction temperature of 51°C, a liquid–solid ratio of 17 mL/g, and an extraction time of 38 min (98). To optimize the extraction of polysaccharides from *C. officinalis* fruits and investigate the impact of various factors on the extractable content of polysaccharides, response surface methodology (RSM) has also been employed. The results indicated that after optimization, the polysaccharide extraction rate was 9.29 ± 0.31% at an extraction temperature of 55°C, ultrasound time of 97 min, pH of 4.2, and a dosage of 2.15% of the complex enzyme (99). The polysaccharides in *C. officinalis* were further purified using DEAE-52 and Sephadex G-100 chromatography, revealing the presence of glucose, arabinose, fucose, xylose, mannose, and rhamnose (100).

The diverse array of bioactivities and pharmacological effects exhibited by *C. officinalis* polysaccharides has captured the attention of researchers both domestically and internationally. These polysaccharides offer a range of benefits, including immunomodulatory, cardiovascular protective, antioxidant, hypoglycemic, and anticancer properties. In a study utilizing an acute myocardial infarction (AMI) rat model to investigate the impact of *C. officinalis* polysaccharides on AMI rats, the results, compared with the model group, revealed an increase in left ventricular systolic pressure (LVSP) and a significant reduction in the myocardial infarction area. This suggests that *C. officinalis* polysaccharides play a crucial role in enhancing cardiac function and reducing the size of myocardial infarcts, possibly through the activation of the GSK-3β signaling pathway, imparting a cardioprotective effect (101). Furthermore, research has explored the antibacterial and antioxidant properties of polysaccharides derived from *C. officinalis* leaves. The isolated polysaccharide fractions demonstrated robust antioxidant activity by effectively scavenging DPPH, hydroxyl, and superoxide anion radicals. Additionally, they exhibited antibacterial efficacy against *Salmonella typhimurium*, *Bacillus subtilis*, *Escherichia coli*, and *Staphylococcus aureus* (102).

### 5.4 Amino acids

The abundance of amino acids in *C. officinalis* is noteworthy. Metabolomic analysis conducted in a specific study identified the presence of 17 amino acids in *C. officinalis* fruits, encompassing all seven essential amino acids determined by biological techniques (103). Notably, the amino acid composition, with aspartic acid (Asp), glutamic acid (Glu), and leucine (Leu) being the predominant ones, exhibited pharmacological activity (3). These amino acids play a pivotal role in the metabolic control of glycolipids, kidney strengthening, and liver promotion, contributing to the functional aspects of *C. officinalis* (104, 105). Furthermore, current nutritional research suggests that foods with excessively high amino acid contents may diminish the nutritional value of proteins. Analyzing the amino acid content of *Cornus sativus* using the amino acid ratio coefficient method revealed that the quantity of amino acids in *C. officinalis* is reasonably balanced, aligning with the body’s requirements for effective absorption and utilization of amino acids (106, 107).

## 6 Pharmacology of *Cornus officinalis*

### 6.1 Antioxidant effect

A study was conducted to investigate the antioxidant properties of *C. officinalis* fruits. By measuring its total phenolic and flavonoid content, DPPH radical scavenging rate, and ABTS radical scavenging rate, the study determined that *Cornus officinalis* exhibited good antioxidant properties (108). The IC_50_ values for DPPH and ABTS radical scavenging rates were measured as 99.32 μg/mL and 138.51 μg/mL, respectively. This indicates the ability of *C. officinalis* to effectively scavenge free radicals and exhibit antioxidant activity. In another study focusing on the ethanolic extract of *C. officinalis* and its potential effect on atopic dermatitis (AD), the EC50 values for the DPPH radical scavenging rate, ferric reducing antioxidant capacity, and ABTS radical scavenging rate were measured as 1.82, 10.76, and 0.6 mg/mL, respectively. These findings suggest that the ethanolic extract of *C. officinalis* possesses antioxidant activity, further supporting its potential application in promoting skin health (109). Furthermore, a study investigated the antioxidant capacity of *C. officinalis* extract in protecting against oxidative damage induced by tert-butyl hydroperoxide (t-BHP) in Chang cells. The results demonstrated that *C. officinalis* extract exhibited antioxidant capacity by enhancing cell viability and preventing the generation of reactive oxygen species (110). Additionally, a study evaluated the antioxidant activity of 20 different varieties of *C. officinalis* plants using various photometric methods. The antioxidant activity, measured by the DPPH method (2,2-diphenyl-1-propenyl hydrazide) (μmol Trolox/g), ranged from 5.94 (Kozerog) to 16.56 (Kostia). The ABTS method (2,2-amino-3-ethylbenzothiazoline-6-sulfonic acid) yielded values ranging from 13.560 (Koralovyj Marka) to 33.96 (Semen). The FRAP method (ferric reducing antioxidant capacity) ranged from 8.45 (Koralovyj) to 22.49 (Kostia). These results indicate the presence of antioxidant activity in different varieties of *C. officinalis* (111).

### 6.2 Reproductive effects

*Cornus officinalis* is widely used in Chinese medicine as a tonic and has been recognized for its beneficial pharmacological effects on the reproductive system. It contains bioactive substances that can improve erectile dysfunction, enhance sperm quality, and treat urinary disorders. These effects were found to be safe (112). In a study, a new formulation was developed by combining *C. officinalis* extract with *Psoralea corylifolia* and evaluated for its effectiveness in treating benign prostatic hyperplasia (BPH). The results showed that the combination formulation exhibited superior efficacy compared to finasteride, suggesting its potential as a novel treatment option for BPH (113). Another study investigated the effects of intravenously administered *C. officinalis* extract on diabetic rats (DM rats) at high and low concentrations (30 mg/kg and 15 mg/kg, respectively). After a 12-week treatment, the rats showed improvements in various parameters, including body weight ratio, sperm rate, testosterone levels, luteinizing hormone (LH) and gonadotropin-releasing hormone (GnRH) levels, as well as reduced serum creatinine, urea nitrogen, and urinary protein excretion. Moreover, testicular morphology was also improved. These findings indicate that *C. officinalis* extract effectively ameliorated testicular damage induced by diabetes mellitus (114).

### 6.3 Anti-diabetic effects

The development of diabetes-related pathologies involves various factors, including the formation of advanced glycosylation end products (AGEs), increased flux in the polyol pathway, activation of protein kinase C isoforms, and hexosamine pathway flux. In a study, galloyl glucosides and lignans isolated from *C. officinalis* seeds demonstrated potent inhibitory activity against AGE formation, suggesting their potential for treating diabetes associated with AGEs (115). In a study involving mice with noninsulin-dependent diabetes mellitus (NIDDM), an ethanol extract of *C. officinalis* was orally administered daily, while a control group was established (116). The mRNA and protein expression in skeletal muscle was evaluated using Northern blot and Western blot methods after 1 month of treatment. Compared to the control group, the ethanol extract of *C. officinalis* significantly increased mRNA expression (*p* < 0.01), indicating its ability to promote islet proliferation and postprandial insulin secretion and enhance mRNA and protein expression in NIDDM mice (117). These results suggest that the extract may improve the high-glucose and high-fat conditions associated with type 2 diabetes, offering potential implications for its treatment (116). The secoiridoids present in *C. officinalis* fruit extract were investigated and found to hold promise as a novel approach for preventing or treating diabetes (118). Furthermore, the effect of oleanolic acid, found in *C. officinalis*, on insulin secretion in rats was studied by administering specific levels of oleanolic acid via intraperitoneal injection. The results demonstrated that oleanolic acid effectively lowered plasma glucose levels by promoting increased plasma insulin levels (119). In a comparative study on diabetic rats, the antidiabetic effect of *C. officinalis* extract was evaluated and compared with that of the drug glibenclamide. The results revealed that *C. officinalis* extract significantly reduced blood glucose levels and increased insulin levels, indicating its potential for facilitating diabetes treatment (120).

### 6.4 Anti-inflammatory activity

A study investigating the anti-inflammatory mechanisms of *C. officinalis* extracts in lipopolysaccharide (LPS)-stimulated macrophages and colitis mice demonstrated their ability to modulate the NF-κB and MAPK signaling pathways. By inhibiting the binding of LPS to TLR4 on immune cells, *C. officinalis* extracts exhibited anti-inflammatory activity. In this study, RAW 264.7 macrophages were treated with an ethanol-extracted extract of *C. officinalis* var. koreana Kitam (COE) at various concentrations (0, 50, 100, 200, and 400 μg/mL). Pretreatment with 100, 200, and 400 μg/mL COE significantly reduced lipopolysaccharide (LPS)-stimulated protein kinase B (Akt) phosphorylation (*p* ≤ 0.003), indicating that the inhibition of protein kinase B (Akt), potentially due to COE treatment, attenuated the inflammatory response induced by LPS in RAW 264.7 macrophages (121). Furthermore, four novel phenolic compounds derived from *C. officinalis* fruits displayed inhibitory effects on NO secretion in RAW 264.7 cells and demonstrated some anti-inflammatory activity against RAW 264.7 cells (122). Given the increasing prevalence of obesity worldwide, which often accompanies various inflammatory diseases, a study explored the combination of *C. officinalis* with other herbs to obtain potential anti-inflammatory drugs for obesity-related inflammation (123). Conducting a surgical examination on the knees of male rats with medial meniscus instability (DMM), loganin was subsequently injected into the osteoarthritic gap at concentrations of 30 or 100 μg/mL over a period of 2–3 months. The findings indicated a significant delay in the progression of the disease (124).

### 6.5 Potential protective effects of *Cornus officinalis* on kidney and liver health

The excessive accumulation of extracellular matrix (ECM) in renal cells is a characteristic of chronic kidney disease, with components such as collagen (Col IV), fibronectin (FN), and pro-inflammatory factors (IL-6) being detrimental to renal cells. However, *C. officinalis* has been found to inhibit the secretion of FN and IL-6 induced by high glucose in renal cells, potentially impacting the development of chronic kidney disease. Through pharmacological and chemical research methods, a bioactive compound called 5-methylfurfural (5-HMF) (125, 126) has been extracted from *C. officinalis*. Studies in mice have shown that 5-methylfurfural (5-HMF) can improve acute liver injury and protect human venous epidermal cells from the harmful effects of H_2_O_2_ and glucose, indicating its beneficial effects on liver and kidney protection (127). Additionally, a combination of iridoid glycosides (IGCO) and triterpene acids (TACO) from *C. officinalis*, along with iridoid glycosides (IGRR) from groundnut, may exert a protective effect on the kidneys through synergistic action (128). An iridoid compound present in *C. officinalis* has the potential to improve alcohol-induced intestinal microbial disorders, protect the gastrointestinal tract, and mitigate the hepatic damage caused by alcohol (129). Moreover, in a mouse model, the ethanol extract (ECO) derived from *C. officinalis* fruits exhibited hepatoprotective effects by preventing or mitigating oxidative stress, thereby reducing liver damage induced by acetaminophen (APAP) (130).

Furthermore, the effect of *C. officinalis* extract on human hepatocyte lines (L02) exposed to D-galactosamine (GalN) and tumor necrosis factor-α (TNF-α) injury was investigated. The findings indicate that CIG significantly enhances the viability of L02 cells subjected to GalN/TNF-α injury, highlighting its potential role in the treatment of liver diseases (131).

### 6.6 Antibacterial effect

*Cornus officinalis* extract has demonstrated a significant inhibitory effect on hepatitis C virus protease activity through bioguided distillation (29). In addition, the ethanolic extract of *C. officinalis* has shown varying degrees of inhibition against bacterial strains such as *E. coli*, *Listeria monocytogenes, E. coli* O157:H7, *Staphylococcus aureus*, *Pseudomonas aeruginosa*, and *Salmonella typhi*, as observed in a disk diffusion assay, where it produced inhibition zones ranging from 8.5 to 18.3 mm at 4000 μg/disc (132). Moreover, the use of 1,3-butanediol (1,3-BG) and ethanol (EtOH) as extraction solvents for the seeds of *C. officinalis* resulted in inhibitory activity against *Staphylococcus aureus*, *Propionibacterium acnes*, and *Staphylococcus epidermidis*. Notably, when comparing silver nanoparticles (AgNPs) synthesized using the aqueous extract of *C. officinalis* with AgNPs synthesized using chemical methods, the former exhibited significantly enhanced inhibition of *E. coli* and *S. aureus*, with 20–40 times greater inhibitory ability (133).

### 6.7 Anticancer activity

Cancer, a malignant tumor resulting from abnormal cell proliferation, is primarily caused by genetic abnormalities that lead to uncontrolled and accelerated cell growth. Although modern medicine has made significant advancements in cancer treatment, certain types of cancer, such as triple-negative breast cancer (TNBC), pose challenges due to limited effective treatments and the development of systemic toxicity and acquired tumor resistance during therapy. However, studies investigating the nutritional properties of medicinal herbs have revealed that extracts from *C. officinalis* exhibit inhibitory effects on TNBC cell cultures, leading to cell growth arrest. Another highly fatal cancer, hepatocellular carcinoma (HCC), predominantly affects the liver (134). In experiments using an aqueous extract of *C. officinalis* at a concentration of 100 μg/mL, all three human hepatocellular carcinoma cell lines (HepG2, SK-Hep1, and PLC/PRF/5) showed inhibition according to the cell activity assay (XTT). Furthermore, researchers isolated an active compound called SZYY from the acetone extract of *C. officinalis* leaves and studied its impact on the proliferation and migration of human malignant melanoma cells (A375) and STAT3 signaling. The findings demonstrated that SZYY can inhibit STAT3 signaling, thereby suppressing tumor angiogenesis and exerting anti-A375 activity (135).

### 6.8 Other effects

To investigate the antimenopausal effects of a combined extract of *C. officinalis* and Cockscomb (RF), the extract was administered to ovariectomized (OVX) mice. The study revealed several positive outcomes, including reduced deposition of fatty cells in the liver and abdominal visceral adipose tissue. Furthermore, there was a significant improvement in bone mineral density and content in the mice, indicating the potential of the CO + RF extract to exert anti-obesity and anti-osteoporosis effects (136). Through cytotoxicity assays, reverse transcriptase-polymerase chain reaction (RT–PCR), and Western blot analysis, it was demonstrated that *C. officinalis* effectively inhibits receptor activator-mediated osteoclast differentiation, thereby reducing the risk of fractures associated with osteoporosis (137). Additionally, *C. officinalis* fruit extracts were found to maintain stable calcium homeostasis by preventing excessive calcium accumulation induced by PM2.5 and protecting cells from PM2.5-induced DNA damage. These extracts also exhibited protective effects against oxidative stress in human HaCaT keratinocytes, preventing lipid peroxidation and protein carbonylation caused by PM2.5. Moreover, *C. officinalis* methanol extract (COME) was shown to impact melanin content in Melana cells, with a significant 36.1% increase observed after treatment with 12.5 μg/mL COME. This suggests the potential of COME in treating gray hair and promoting hair growth (138, 139). In a study on the oxidative mutagenic effect of *C. officinalis* on human neutrophils (PMNs), it was found that the extract inhibits the secretion of important chelators, such as IL-8, by human neutrophils and Caco-2 intestinal epithelial cells. This balancing effect on the immune system and epithelial cells is significant (140). Furthermore, the effects of *C. officinalis* extract on rats with cerebral infarction were investigated, revealing a reduction in the infarct area, NO content, NOS activity, and the number of NF-kappaB-positive cells in the cerebral cortex compared to the control group. These findings highlight the potential benefits of *C. officinalis* extract in the treatment of cerebral infarction (141).

## 7 Existing products in the market

*Cornus officinalis* possesses significant nutritional value, as indicated in Table 3. It primarily serves the pharmaceutical industry, where it is processed to form an essential component of drugs used to treat various ailments. As the national economy develops and people’s living standards improve, there is an increasing demand for functional health foods*. C. officinalis* dried fruit pulp is commonly utilized in the production of tea and wine (142, 143). Additionally, *C. officinalis* powder can be used for foot soaks or incorporated into foot patches, offering benefits for individuals with high blood pressure and chronic pharyngitis (144). The applications of *C. officinalis* extend beyond these examples and encompass the production of health drinks, jams, preserved fruits, canned fruit pulp, and more (145, 146). Moreover, studies have explored the preparation of *C. officinalis*, wolfberry, and danfeng peony as raw materials, with high-quality cooking wine as the base material, to create a flavonoid-rich and antioxidant-packed wine product through appropriate brewing techniques (147).

*Cornus officinalis* harbors a diverse range of endophytic bacteria (148). Previous research has focused on examining the beneficial properties of *C. officinalis* fruit fermentation, including acid and bile salt tolerance, antibacterial activity, self-aggregation, and cholesterol-lowering effects. From these fermented *C. officinalis* fruits, probiotic lactic acid bacteria (LAB) with desirable characteristics, such as acid and bile salt tolerance, antimicrobial activity, self-aggregation, and cholesterol-lowering capacity, have been identified. One such example *is Lactobacillus plantarum*. *Lactobacillus plantarum* is known for its positive impact on health, including immunomodulatory functions, maintenance of intestinal flora balance, promotion of nutrient absorption, alleviation of lactose intolerance, and inhibition of tumor cell formation. The LAB discovered from *C. officinalis* fermentation hold potential as probiotic candidates (148).

## 8 Conclusion and future perspectives

For centuries, the fruits of *Cornus officinalis* have been esteemed for their rich nutritional profile. This recognition is primarily attributed to the presence of diverse active components, including polyphenols, flavonoids, irises, anthocyanins, organic acids, and various bioactive compounds. Consequently, *C. officinalis* holds significant potential in promoting human health and combating various diseases, such as diabetes, kidney and liver disorders, and cancer. However, in-depth studies on the pharmacodynamics and pharmacotoxicology of *C. officinalis* fruits and their derived bioactive compounds are imperative to ascertain their efficacy and safety for human consumption.

The aforementioned insights collectively underscore extensive research on *C. officinalis*, revealing its advantageous effects on the reproductive system, anticancer properties, antidiabetic effects, and hypolipidemic effects. Additionally, noteworthy exploration has been undertaken to create novel food products with exceptional flavors that contribute to overall health. In Chinese culture, it has been a longstanding tradition to incorporate *C. officinalis* into porridge, wine-making, and soup preparation alongside other nutritional supplements. In recent years, the food industry has embraced the utilization of *C. officinalis* to develop healthy options, jams, wines, and jellies. Researchers have even introduced a *C. officinalis* complex crystal solid drink, with *C. officinalis* as the primary ingredient, sucrose as a sweetener, and other fruit juice concentrates as flavor enhancers. Looking ahead, there is a promising outlook for the development of additional compounds with remarkable nutritional value.

## Data availability statement

The original contributions presented in the study are included in the article/supplementary material, further inquiries can be directed to the corresponding authors.

## Author contributions

WD: Supervision, Writing – review & editing. YL: Investigation, Writing – original draft. YG: Supervision, Writing – review & editing. JC: Writing – review & editing. HA: Supervision, Writing – review & editing. MK: Supervision, Writing – review & editing. CP: Supervision, Writing – review & editing. JP: Resources, Supervision, Writing – review & editing. AE-A: Supervision, Writing – review & editing.

## Footnotes

^1^www.webofscience.com

^2^https://bibliometric.com
